# The Roles of RAC1 and RAC1B in Colorectal Cancer and Their Potential Contribution to Cetuximab Resistance

**DOI:** 10.3390/cancers16132472

**Published:** 2024-07-06

**Authors:** Claudia C. Wahoski, Bhuminder Singh

**Affiliations:** 1 Program in Cancer Biology, Vanderbilt University, Nashville, TN 37232, USA; 2 Department of Medicine, Vanderbilt University Medical Center, Nashville, TN 37232, USA

**Keywords:** colorectal cancer, cetuximab, drug resistance, RAC1, RAC1B

## Abstract

**Simple Summary:**

Cetuximab is a treatment widely used to treat advanced, metastatic colorectal cancer (CRC), and it works by blocking epidermal growth factor receptor signaling. Unfortunately, patients inevitably develop resistance to cetuximab. The most common resistance mechanisms are well established, but there are still patients that become resistant to cetuximab due to unknown mechanisms. The small guanosine triphosphatases (GTPases) RAC1 and RAC1B have been shown to contribute to CRC progression, but their role in cetuximab resistance is unclear. This review highlights the known cetuximab resistance mechanisms and summarizes how RAC1 and RAC1B could contribute to these resistance mechanisms to propose RAC1 and RAC1B as additional therapeutic targets that could increase the efficacy of cetuximab.

**Abstract:**

Colorectal cancer (CRC) is one of the most diagnosed cancers and a leading contributor to cancer-related deaths in the United States. Clinically, standard treatment regimens include surgery, radiation, and chemotherapy; however, there has been increasing development and clinical use of targeted therapies for CRC. Unfortunately, many patients develop resistance to these treatments. Cetuximab, the first targeted therapy approved to treat advanced CRC, is a monoclonal antibody that targets the epidermal growth factor receptor and inhibits downstream pathway activation to restrict tumor cell growth and proliferation. CRC resistance to cetuximab has been well studied, and common resistance mechanisms include constitutive signal transduction through downstream protein mutations and promotion of the epithelial-to-mesenchymal transition. While the most common resistance mechanisms are known, a proportion of patients develop resistance through unknown mechanisms. One protein predicted to contribute to therapy resistance is RAC1, a small GTPase that is involved in cytoskeleton rearrangement, cell migration, motility, and proliferation. RAC1 has also been shown to be overexpressed in CRC. Despite evidence that RAC1 and its alternative splice isoform RAC1B play important roles in CRC and the pathways known to contribute to cetuximab resistance, there is a need to directly study the relationship between RAC1 and RAC1B and cetuximab resistance. This review highlights the recent studies investigating RAC1 and RAC1B in the context of CRC and suggests that these proteins could play a role in resistance to cetuximab.

## 1. Introduction

Colorectal cancer (CRC) is predicted to be the second leading cause of cancer-related deaths for both men and women combined in 2024 [1]. It is also expected to be the third most frequently diagnosed cancer in men and women, with over 150,000 new cases predicted in the United States in 2024 alone [1]. Furthermore, since 1995, there has been a 9% increase in the number of younger people diagnosed with CRC, where now 20% of CRC diagnoses are in people under 54 years old, which is an emerging trend in recent years [2]. The five-year survival rate for CRC negatively correlates with the stage at diagnosis where distant, metastasized disease has a lower five-year relative survival rate (15%) relative to localized disease (90%) [3]. While the overall incidence of CRC in the United States has declined from around 60 people per 100,000 in 1975 to 31 per 100,000 people in 2020, the relative five-year survival rate at all stages of disease has not significantly improved (63% in 2000 compared to 65% in 2015), suggesting that there has not been substantial improvement in prolonging the life of patients with CRC [4,5]. These data suggest that better understanding of CRC, treatment options, and the effects of these treatments is needed to improve patient outcomes.

### 1.1. Colorectal Cancer Treatment—Surgery, Chemotherapy, and Targeted Therapies

Patients with local and resectable tumors generally receive surgery as a first course of treatment. Surgery involves removing the tumor and any tumor-draining lymph nodes, along with other tumor-associated tissue. Following surgery, patients receive chemotherapy and radiotherapy to eliminate the remaining cancer cells. Chemotherapy given for CRC generally includes 5-fluorouracil (5-FU), irinotecan, oxaliplatin, and capecitabine, either as single agents or in combination [6]. While chemotherapy can be an effective treatment option, there are significant limitations including systemic toxicity, primary and acquired resistance, and low tumor specificity, thus, there is a need for alternative therapies.

Targeted therapy is another treatment option that employs a small molecule or antibody targeting a specific molecule that is generally unique to the tumor cells or is present at higher levels in the tumor relative to normal tissue. These drugs can be administered as single agents or in combination with chemotherapy, and they are generally administered after surgical resection of the primary tumor. The approved monoclonal antibodies for CRC include epidermal growth factor receptor (EGFR)-targeting antibodies such as cetuximab and panitumumab and the vascular endothelial growth factor (VEGF)-targeting antibody bevacizumab. Other targeted therapies like small molecules are approved to treat CRC, such as mutant BRAF inhibitor encorafenib and the multi-kinase inhibitor regorafenib [6]. Targeted therapies represent a more direct way to kill cancer cells while having limited effects on normal cells compared to chemotherapy.

### 1.2. Cancer Treatment Resistance

While cancer treatments are continuously being developed with increasing specificity and efficacy, most patients develop resistance to therapy. Resistance occurs in part due to tumor heterogeneity, where a diverse array of cells with various phenotypes exist within the same tumor [7]. Treatment with targeted therapies is likely to kill only a subpopulation of cells within the tumor, thus selecting for other subpopulations that do not respond to therapy and leaving a tumor that can have multiple mechanisms of resistance to the therapy [7]. Cancer resistance mechanisms can be classified depending on their origin (de novo/intrinsic/primary vs acquired resistance) and underlying cause (genetic or nongenetic). Primary resistance refers to cell populations that are resistant to therapy before any treatment has begun. Acquired resistance occurs when a tumor is treated with a specific therapy for a prolonged period and shows an initial clinical response but eventually relapses. Tumors with acquired resistance can have cells that adapt, persist, and evolve to grow under the presence of the drug, or the resistant cells already existed within the tumor [8].

Resistance to targeted therapy is primarily driven by genetic changes such as mutations within the gene targeted by the drug or other genes in the pathway, gene amplification, gene deletion, or chromosomal translocation resulting in oncogenic gene fusions [9]. Genetic changes are a main contributor to drug resistance in general and are more likely to be identified as they can easily be tested for in the clinic. However, some patients who do not respond to therapy do not show a genetic cause of resistance, thus nongenetic mechanisms have been increasingly studied. Nongenetic mechanisms of resistance refer to changes in gene expression or epigenetic gene regulation, resulting in increases or decreases in mRNA levels of a particular gene, activation of complementary receptors and signaling pathway crosstalk, increases in drug efflux, and alterations in cell metabolism [10,11]. Nongenetic resistance also allows for phenotypic plasticity where cancer cells can change characteristics rapidly or become tolerant to treatment [12,13]. Identifying the common nongenetic mechanisms, either in isolation or in addition to genetic causes of resistance, can help identify other druggable pathways that can increase overall treatment efficacy.

### 1.3. Epidermal Growth Factor Receptor and Cetuximab

EGFR is a receptor tyrosine kinase (RTK) that is overexpressed in up to 80% of CRC cases, indicating its potential as a therapeutic target [14]. EGFR signaling in normal epithelial cells is responsible for controlling cell growth, proliferation, survival, migration, and angiogenesis [15,16]. In cancer cells, aberrant EGFR activation through receptor overexpression or mutation, or constitutive pathway activation through mutations in downstream signaling components leads to uncontrolled growth, migration, and angiogenesis, promoting tumorigenesis [16].

Cetuximab is a mouse-derived monoclonal IgG1-type chimeric antibody that targets the extracellular domain III of EGFR to prevent ligand binding, receptor dimerization, and subsequent receptor phosphorylation and activation [17,18,19,20]. Cetuximab also promotes EGFR internalization and degradation, induces cell cycle arrest and apoptosis, decreases angiogenesis and metastasis, and mediates antibody-dependent cellular cytotoxicity [21,22]. Cetuximab was approved by the Food and Drug Administration (FDA) in 2004 after it was shown to increase patient response rates and delay median progression time when administered in combination with chemotherapy, and it was the first targeted therapy approved for CRC [23]. Currently, cetuximab is approved as a first- or second-line therapy alone or in combination with chemotherapy only for patients with advanced, metastatic left-sided tumors that have the wild type forms of the EGFR downstream proteins Kirsten rat sarcoma viral oncogene homolog (KRAS) and v-raf murine sarcoma viral oncogene homolog B1 (BRAF), as it has been shown that oncogenic KRAS/BRAF mutations decrease the efficacy of cetuximab through constitutive downstream pathway activation independent of the receptor [24,25,26,27]. This means that cetuximab is limited in use for patients diagnosed with distant disease, making up approximately 23% of patients, and approximately 35–45% of tumors have KRAS mutations, and another 10% have BRAF mutations, limiting cetuximab use to the remaining 45% of patients [4,5,28].

In addition to cetuximab, another monoclonal antibody, panitumumab, is FDA approved to treat KRAS/BRAF wild-type metastatic CRC as a first- or second-line therapy. Panitumumab is a humanized monoclonal IgG2-type antibody that binds to the extracellular domain III of EGFR and has similar mechanisms of action to cetuximab [29,30]. Panitumumab binds to a different location on EGFR compared to cetuximab, which could indicate its possible usage as an alternative treatment if patients have adverse reactions to cetuximab or after cetuximab failure due to resistance from EGFR mutations [31,32]. In particular, patients in the southeastern region of the United States develop severe hypersensitivity reactions to cetuximab due to the presence of IgE antibodies against galactose-α-1,3-galactose, which is a modification found on cetuximab [33].

Recent clinical trials have shown that cetuximab and panitumumab can be effective in patients with specific KRAS and BRAF mutations when used in combination with other inhibitors. Adagrasib and sotorasib are KRAS G12C inhibitors that show an increased effect when used in combination with cetuximab or panitumumab, respectively [34,35]. Additionally, patients with mutated BRAF (V600E) can benefit from triplet combination therapy with the BRAF V600E inhibitor encorafenib, cetuximab, and the inhibitor binimetinib targeting the downstream signaling protein MEK [36]. Using cetuximab in combination with these targeted inhibitors is important to prevent activation of feedback loops that are mediated upstream by EGFR [37]. Other therapeutic combinations with cetuximab are also being investigated, including the use of HER2 inhibitors and immunotherapy in combination with cetuximab, although these combinations are not FDA-approved [27,38]. These clinical trials suggest that targeted therapies can be used in combination to provide better prognosis for patients with advanced CRC. These trials also provide evidence for expanding the use of cetuximab to include more patients, supporting research into combination therapies and new drug targets.

### 1.4. CRC Resistance to Cetuximab

**Figure 1:**
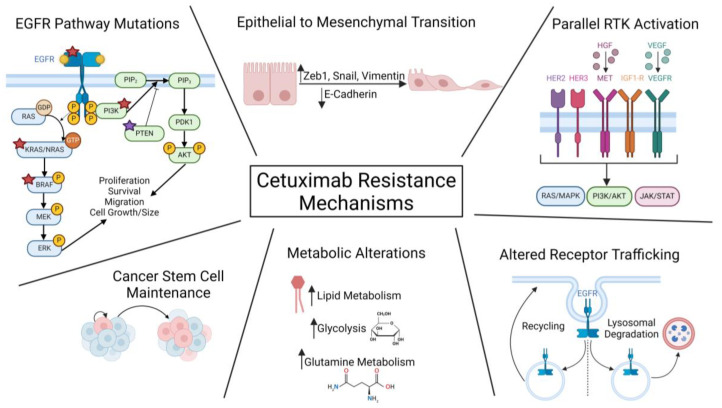
Genetic and nongenetic mechanisms of CRC resistance to cetuximab. Briefly, genetic mechanisms of resistance include activating mutations in KRAS, BRAF, PI3K, and EGFR (red stars) and loss of function mutations in PTEN (purple star). These mutations in the EGFR signaling pathway lead to constitutive signal activation independent from EGFR activation, and this ultimately leads to increased cell proliferation and survival. Other resistance mechanisms include nongenetic modes of resistance, including the epithelial-to-mesenchymal transition through increased expression of EMT transcription factors like Snail and Zeb1, activation of parallel receptor tyrosine kinases (RTK), and altered EGFR trafficking. These mechanisms are discussed more in depth in Section 4.1, Section 4.2, Section 4.3, and Section 4.4. Other resistance mechanisms that are not as well studied but nonetheless play a role in cetuximab resistance include maintenance of cancer stem cells and altered cellular metabolism. Figure created in Biorender.com.

Resistance to EGFR-targeting therapies is almost universal in CRC. The most common resistance mechanisms originate from genetic alterations and include activating mutations in EGFR downstream pathway components KRAS, BRAF, and phosphatidylinositol-4,5-bisphostate 3-kinase (PI3K) catalytic subunit alpha (PIK3CA) (Figure 1) [39,40]. KRAS- and BRAF-mutated tumors have intrinsic primary resistance to cetuximab, but mutations also arise following cetuximab treatment as a form of acquired resistance [39,40,41]. Recent studies have shown that treatment of CRC with EGFR inhibitors is more likely to lead to KRAS/BRAF mutations compared to general cytotoxic chemotherapy, suggesting that mutations in these key proteins should be expected when treating patients, further amplifying the need for alternative and combination therapies [42]. Other mechanisms of resistance to cetuximab include nongenetic alterations such as parallel RTK upregulation leading to activation of signaling pathways, metabolic alterations, cancer stem cell maintenance, and initiation of the epithelial-to-mesenchymal transition (EMT) (Figure 1) [43,44]. EMT is a well-known phenomenon in cancer, but direct evidence for EMT as a cetuximab resistance mechanism in CRC is limited. Proteins contributing to EMT in CRC include the small guanosine triphosphatases (GTPases) Rac family small GTPase 1, previously known as Ras-related C3 botulinum toxin substrate 1 (RAC1), and its alternative splice isoform RAC1B.

As members of the RHO family of small GTPases, which includes RhoA and Cdc42, RAC1 and RAC1B are involved in cytoskeleton rearrangement, cell proliferation, and cell migration [45]. RAC1 and RAC1B have been shown to contribute to CRC by playing a role in EMT, cell migration, invasion, metastasis, survival against chemotherapy, escape from cellular senescence, and altering cellular metabolism by regulating several aspects of CRC cell signaling and morphology [45,46,47,48]. RAC1 and RAC1B are overexpressed in CRC and associated with worse prognosis compared to tumors that do not have RAC1 or RAC1B overexpression, suggesting there is a role for these proteins in CRC biology and tumor progression [49]. There are also reports for the role of RAC1 and RAC1B in chemotherapy and targeted therapy resistance in cancers such as melanoma, lung cancer, breast cancer, and thyroid cancer, and this has been reviewed elsewhere [45,47,50,51].

While RAC1 and RAC1B have not been investigated specifically in the context of cetuximab resistance in CRC, RAC1 and RAC1B biology and signaling pathways suggest that their overexpression could contribute to cetuximab resistance, presenting a new target for combination therapy. This review focuses on resistance mechanisms of CRC against cetuximab and discusses the possibility that RAC1 and RAC1B could play a role in the development of cetuximab resistance through these mechanisms.

## 2. RAC1 and RAC1B Structure and Expression

### 2.1. RAC1 Structure and Function

**Figure 2:**
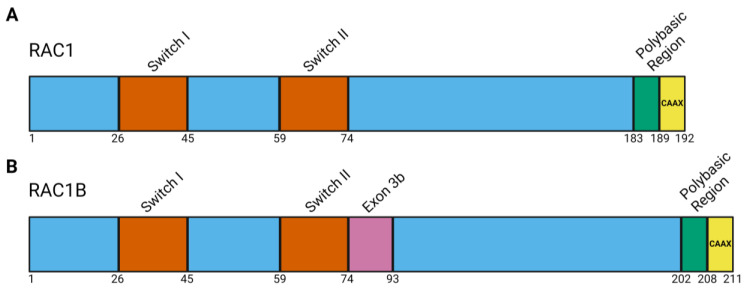
RAC1 and RAC1B protein domains. (**A**) Schematic of RAC1 protein domains highlighting the switch I and switch II domains that direct the conformational change to facilitate GTP binding and subsequent effector protein activation. (**B**) Schematic of RAC1B protein domains including exon 3b, resulting in an additional 19 amino acids following the switch II domain. The conformational changes of RAC1 and RAC1B driven by the switch I domain directs interactions with downstream effector proteins leading to pathway activation. Following the switch II domain is a long C terminal region, also called the hypervariable region. The hypervariable region is followed by a polybasic region that is then followed by a CAAX motif (C represents cysteine, A represents an aliphatic residue, and X represents any amino acid). The CAAX motif in RAC1 and RAC1B is CLLL (where L is leucine). The polybasic region and CAAX motif direct RAC1 intracellular localization through the prenylation post-translational modification, specifically geranylgeranylation [55]. The polybasic region also serves as a nuclear localization signal that directs RAC1 to the nucleus where it can impact gene transcriptional programs [56]. Figure created in Biorender.com.

The RAC1 protein sequence is 192 amino acids long and contains the switch I and switch II domains that direct the conformational changes responsible for protein activation and guanosine diphosphate (GDP) or guanosine triphosphate (GTP) binding (Figure 2A, Table 1) [50,52]. The conformational changes of RAC1 driven by the switch I domain directs interactions with downstream effector proteins, leading to pathway activation. The switch II domain serves as the binding site for guanine nucleotide exchange factors (GEFs) [45]. RAC1 at the plasma membrane is defined as active, but the distribution of RAC1 at the membrane, in the cytoplasm, or in the nucleus is cell-cycle dependent and constantly changing [53,54]. All locations impact RAC1 function and downstream signal activation.

GTPases act as molecular switches as they alternate between an inactive, GDP-bound state and an active, GTP-bound state. RAC1-GDP has a closed conformation rendering it unable to interact with and activate downstream effectors. To become active, GEFs bind to RAC1 and facilitate the exchange from GDP to GTP. RAC1 GEFs include TIAM1, VAV, and TRIO, and these proteins can become activated in response to RTK activation [61]. Active GTPases can execute effector functions including binding and activating other proteins, allowing for effector protein phosphorylation, and nuclear localization to activate gene transcription. GTPase-activating proteins (GAPs) negatively regulate GTPases by promoting the hydrolysis of GTP to GDP. Another mechanism of RAC1 negative regulation is the sequestration of inactive RAC1 in the cytoplasm by guanine nucleotide-dissociation inhibitors (GDIs), thus, cytoplasmic RAC1 is associated with the inactive form.

### 2.2. RAC1B Protein Structure

In addition to the canonical RAC1 protein, there is an alternative splice isoform called RAC1B resulting from an RNA transcript that includes 57 nucleotides between codons 75 and 76, leading to 19 amino acids being added to the RAC1 protein (Figure 2B, Table 1) [57]. This exon, termed exon 3b, immediately follows the switch II domain of RAC1 and was discovered by investigating RAC1 expression in human and fetal tissues, as well as intestinal tumor samples [57]. Around the same time, RAC1B was also discovered to be expressed in breast epithelial tissue and breast tumor tissue [58].

Compared to RAC1, RAC1B has increased GDP to GTP exchange that is independent of GEF activity, and it has been characterized as a self-activating, constitutively active RAC1 isoform [62,63]. Increased activity and downstream signaling activation are likely due to the increased flexibility of the switch I and switch II domains, promoting an open conformation and increased binding of GTP [63]. In vitro analysis of RAC1B activity also shows that RAC1B has impaired GTP hydrolysis, thus prolonging the RAC1B-GTP state and facilitating the activation of downstream effectors [62]. Furthermore, RAC1B cannot interact with RHO-GDIs that would keep RAC1B inactive in the cytoplasm, thus RAC1B is more often active in the cell [64]. Collectively, these characteristics lead to more active RAC1B in the cell available to execute downstream effector functions.

### 2.3. Regulation of RAC1 and RAC1B Isoform Expression

Isoform expression of RAC1 and RAC1B is regulated by differential expression and activation of splicing factors serine/arginine rich splicing factor 1 (SRSF1, also known as SF/AF2), or serine/arginine rich protein 20 (SRp20, also known as SRSF3) that either favor the inclusion or exclusion of exon 3b, respectively [65]. Cellular signaling pathways can modulate SRSF1 and SRSF3 levels where cells with high WNT signaling favor RAC1 expression through increased SRSF3 and cells with high EGFR signaling favor RAC1B expression through SRSF1 expression and phosphorylation [65,66,67]. EGFR signaling through epidermal growth factor (EGF) binding also promotes the ubiquitination and inhibition of heterogeneous nuclear ribonucleoprotein (hnRNP) A1 that promotes exon 3b exclusion [68,69]. The RNA-binding protein epithelial splicing regulatory protein 1 (ESRP1) is increased in CRC cells and is positively correlated with RAC1B mRNA and protein levels [70].

## 3. RAC1 and RAC1B in Colorectal Cancer and Drug Resistance

### 3.1. RAC1 in Colorectal Cacner

The role of RAC1 in the intestinal epithelium and normal physiological cell functions have been previously reviewed [45,47,50,51,71]. In the normal intestinal epithelium, the main function of RAC1 is to maintain epithelial cell polarity and the integrity of tight junctions in the crypt where WNT signaling is high [71,72]. RAC1 is involved in the regulation of cell migration and wound healing in response to damage and inflammation in the normal intestinal epithelium through EGF-induced EGFR activation [73]. Furthermore, RAC1 maintains proper intestinal barrier integrity by regulating cell extrusion in the inflamed intestine [74]. RAC1 signaling increases intestinal stem cell adhesion through the receptor Leucine-rich repeat-containing G protein-coupled receptor 5 (LGR5), which is highly expressed in the cell population and potentiates WNT signaling [72]. The activation of WNT signaling through loss of function mutations or truncations in the protein adenomatous polyposis coli (APC) is an early event in the development of CRC [75]. One study suggests that RAC1 is critical for CRC tumorigenesis following loss of APC, specifically allowing for LGR5+ cell expansion and proliferation [76]. Further investigation into RAC1 in these cells showed that RAC1 drives the production of reactive oxygen species (ROS) and activating nuclear factor kappa B (NFκB) signaling to facilitate tumor progression [76]. The relationships between WNT signaling and RAC1 signaling in the intestinal crypts suggests that RAC1 plays a role in the development of CRC.

RAC1 overexpression was first identified to play a role in cancer progression in breast cancer, and investigations of RAC1 overexpression in other cancer types soon followed [58]. RAC1 expression has been found to be correlated with worse prognosis for many cancers [77]. Specifically for CRC, RAC1 overexpression is correlated with worse overall survival, advanced disease stage, and metastasis [77,78,79]. In cancer, RAC1 can be overexpressed or mutated to become constitutively active, but RAC1 is more likely to be overexpressed in CRC [71,80]. Studies of RAC1 in orthotopic mouse models show that RAC1 accelerates tumorigenesis and progression, and the lack of RAC1 expression suppresses tumor formation [81]. This provides further evidence that RAC1 plays a role in CRC progression.

There is ongoing research investigating the role of RAC1 in CRC, especially with respect to RAC1 regulatory proteins. For example, the RAC1 GEFs dedicator for cytokinesis 4 (DOCK4) and DOCK7 play a role in CRC, where DOCK4 could represent a new CRC biomarker for immune infiltration, and DOCK7 promotes CRC metastasis through secretion in macrophage-derived extracellular vesicles that activate RAC1 in CRC tumor cells [82,83]. Moreover, methylation of the RAC1 GEF TIAM1 can activate RAC1 signaling, which promotes CRC metastasis [84]. This suggests that inhibiting RAC1 through its regulatory proteins like GEFs could be a therapeutic option, as discussed later in this review. Other recent studies of RAC1 in CRC uncover the impact of RAC1 overexpression on cellular metabolism, where RAC1 promotes proliferation, migration, and invasion of CRC cells through SRY-box transcription factor 9 (SOX9), and SOX9 upregulates genes involved in glycolysis [85]. Altered cellular metabolism and increased glycolysis are well-known characteristics of cancer cells, and the association of RAC1 with altered metabolism in CRC could provide another CRC treatment avenue.

### 3.2. RAC1B in Colorectal Cancer

RAC1B is found to be expressed almost exclusively in tumor tissue, suggesting it plays an important role in cancer initiation and progression. The initial investigation into the role of RAC1B in cancer cell biology and tumorigenesis showed that RAC1B overexpression could transform NIH T3T mouse fibroblasts into malignant cells and increase cell-cycle progression [86]. RAC1B promotes cell survival by upregulating anti-apoptotic proteins including AKT2 and MCL1 and upregulating anti-apoptosis pathways such as JNK2/c-JUN/cyclin D1 [87]. These proteins also impact RAC1B isoform expression as depletion of AKT2 decreases RAC1B levels, thus, initiating a feedback loop regulating RAC1B expression [67]. Specifically in CRC, increased cell survival and cell-cycle progression in cells that overexpress RAC1B are due to increased NFkB signaling [88,89,90]. In vivo studies using mice that overexpressed RAC1B in addition to APC loss (*Apc*^min^) showed that RAC1B facilitates the formation of tumors, but RAC1B overexpression alone without the cooperation of APC loss is not sufficient to initiate and drive tumorigenesis [91]. Moreover, *Apc*^min^ mice with RAC1B overexpression had decreased tumor-free survival compared to mice with RAC1B deletion. RAC1B depletion increases apoptosis and decreases cell-cycle progression, indicating that CRC cells depend on RAC1B for survival [89].

Following the initial discovery of RAC1B in CRC tumor samples, clinical investigations found that RAC1B expression in CRC tumors is significantly associated with BRAF mutation status, where approximately 80% of BRAF-mutated CRC tumors also overexpress RAC1B; however, tumors that do not have BRAF mutations can also overexpress RAC1B [92]. The analysis of clinical trial data suggests that RAC1B overexpression is correlated with negative prognosis in patients with metastatic CRC treated with leucovorin calcium (folinic acid), fluorouracil, and oxaliplatin (FOLFOX), and capecitabine and oxaliplatin (XELOX) chemotherapy regimens [93]. Interestingly, analysis of The Cancer Genome Atlas (TCGA) patient data showed that increased RAC1B expression is significantly correlated with advanced stage CRC tumors, decreased disease-free survival, and decreased overall survival [94]. These data also suggest that RAC1B expression status could be used to predict prognosis in patients that have wild-type KRAS and BRAF, as these patients had lower overall survival and progression-free survival compared to patients that did not have RAC1B overexpression.

Recent studies focused on RAC1B in the colon have moved toward the immunological impacts of RAC1B overexpression, and the influence of RAC1B on the tumor microenvironment. An earlier study showed that RAC1B expression is increased in patients with an inflamed colonic mucosa [95]. Using an in vivo model of colon inflammation, one group showed that the resolution of colitis involved RAC1B-driven cell proliferation, migration, and increased ROS production [91]. These processes are involved in intestinal healing after inflammation, suggesting RAC1B interacts with the immune response in epithelial cells. Interestingly, ibuprofen, a nonsteroidal anti-inflammatory drug, decreases RAC1B expression in CRC cells with BRAF mutations [96]. Reduced RAC1B expression after ibuprofen treatment occurs through decreased SRSF1 nuclear translocation, thus providing a potential therapeutic option for patients with intestinal inflammation that could promote CRC development [96]. With respect to the tumor microenvironment, it has been shown that cancer-associated fibroblasts and pro-tumorigenic macrophages secrete IL-6 that triggers STAT3 activation in CRC cells, and this leads to an increase in RAC1B expression in the cancer cells [97].

### 3.3. Comparison of RAC1 and RAC1B in CRC

Compared to RAC1, RAC1B has been shown to activate different signaling pathways that can contribute to differences in cancer cell phenotypes if the cells overexpress RAC1 or RAC1B (Table 2). For example, RAC1B cannot activate p21 activated kinase (PAK1) or JNK, induce lamellipodia formation, or interact with RHO-GDI, which are all common RAC1 effector functions [63,64,86]. Instead, RAC1B binds to the atypical GEF SmgGDS, receptor for activated kinase 1 (RACK1), and p120 catenin that are involved in cell motility, adhesion, and gene transcription [98]. On the other hand, similar to RAC1, RAC1B can stimulate ROS production that promotes the epithelial-to-mesenchymal transition [99,100]. Overall, RAC1 and RAC1B have similar effects on cancer cell phenotypes but utilize different pathways, suggesting they cannot substitute for each other, and both proteins should be investigated in the context of cancer.

Both RAC1 and RAC1B can stimulate WNT signaling and WNT target gene transcription. RAC1 can bind to activated β-catenin and TCF4 to then increase gene transcription. RAC1B can bind to dishevelled and β-catenin to activate WNT signaling and downstream target gene activation, and deletion of RAC1B has been shown to decrease WNT pathway genes [94,101]. Overall, the activation of WNT target genes decreases CRC cell adhesion, thus facilitating tumor progression [56]. Interestingly for RAC1B, the activation of WNT target genes introduces a negative feedback loop as increased WNT target gene expression decreases the inclusion of RAC1 exon 3b, as described above.

RAC1 and RAC1B have both been shown to physically interact with or be located proximal to EGFR and downstream EGFR signaling components [94]. RAC1 can be activated by EGFR signaling, and it can also act as an activator of EGFR downstream effectors, emphasizing the relationship between these pathways [102,103]. With respect to RAC1B, in vitro and in vivo experiments revealed that RAC1B deletion in colorectal cancer decreased EGFR phosphorylation and downstream pathway activation, suggesting RAC1B plays a significant role in EGFR signaling [94]. Recent studies have suggested that in CRC, there is crosstalk between WNT and EGFR/MAPK signaling that cooperates to facilitate tumorigenesis [94,104]. One group found that inhibition of EGFR signaling also alters expression of WNT-activated genes [94]. Based on the evidence presented here, RAC1 and RAC1B could facilitate this cross-talk and provide a target for CRC treatment.

**Table 2:** Summary of the roles of RAC1 and RAC1B in normal intestinal epithelium and colorectal cancer.

Feature	RAC1	RAC1B	Ref.
Roles in Normal Intestinal Epithelium	Maintain epithelial cell polarity, tight junction integrity, and stem cell adhesion, regulate migration and wound healing, regulate cell extrusion,	N/A RAC1B is predominantly expressed in tumor tissue, thus a role in normal tissue is unknown	[46,71]
Roles in CRC	Overexpressed in CRC LGR5+ stem cell expansion following APC loss; ROS production; NFκB signaling; accelerate tumorigenesis and tumor progression; promote cell proliferation, migration, and invasion; alter cellular metabolism; promote EMT	Promote cell survival and decrease apoptosis, increase cell cycle progression and cell proliferation, NFκB signaling, ROS production, mediate BRAF(V600E)-oncogene-induced senescence, promote EMT	[46,71]
Correlation with Patient Outcomes	Increased expression correlated to worse overall survival, advanced disease stage, metastasis Expression in liver metastases higher than primary tumor	Expression related to worse overall survival, advanced disease stage, and worse disease-free survival	[77,78,79,93,94]
Overexpression sufficient to initiate tumorigenesis?	Yes	No	[76,91]
Roles in CRC Therapy Resistance	GAP ARHGAP17 expression increases CRC sensitivity to 5-FU	Expression promotes 5-FU and oxaliplatin resistance through NFκB signaling	[90,105]
Mechanisms for RAC1/RAC1B targeting for combination therapies	GEF-interaction inhibition, GTP-binding inhibition, downstream protein interaction inhibition, post-translational modification inhibition	GEF-interaction inhibition, GTP-binding inhibition, downstream protein interaction inhibition, post-translational modification inhibition	[51]

### 3.4. RAC1 and RAC1B Contribution to CRC Chemotherapy Resistance

The role of RAC1 in CRC chemotherapy resistance is understudied. However, one report suggests expression of the RAC1 GAP ARHGAP17, which negatively regulates RAC1 activation and signaling and increases CRC cell sensitivity to 5-FU treatment [105]. This suggests that proteins regulating RAC1 activity in addition to RAC1 itself can be involved in treatment resistance.

On the other hand, the role of RAC1B in therapy resistance has been directly investigated. As mentioned previously, RAC1B overexpression in CRC cell lines increases cell survival by inhibiting apoptosis and increases cell viability and proliferation by activating NFκB signaling [90]. In this same study, RAC1B overexpression led to 5-FU and oxaliplatin resistance and knockdown of RAC1B or inhibiting RAC1B with the small molecule GYS32661 sensitizes cells to chemotherapy treatment [90]. Interestingly, RAC1B overexpression was seen after treating cells with chemotherapy, suggesting that RAC1B overexpression is a response to treatment and could be a form of acquired resistance.

While studies on how RAC1 and RAC1B contribute to chemotherapy resistance and targeted therapy resistance in CRC are scarce, RAC1 has been shown to be involved in therapy resistance in other cancers such as breast cancer, melanoma, prostate cancer, pancreatic cancer, and others [106,107,108,109,110,111,112,113,114]. RAC1 and RAC1B in breast cancer therapy resistance have been studied recently, where RAC1 can contribute to chemotherapy resistance by altering metabolic pathways like the pentose phosphate pathway, and RAC1B has been shown to maintain stem cell populations that confer chemo-resistance [106,115]. Both metabolic alterations and stem cell maintenance are seen in CRC resistance to cetuximab, as described above. The current research on the roles of RAC1 and RAC1B in therapy resistance in a variety of cancers suggests that research into the relationship between RAC1, RAC1B, and cetuximab resistance in CRC should be investigated. In the sections below, we highlight the known signaling pathways and protein interactions of RAC1 and RAC1B in the context of known cetuximab resistance mechanisms.

## 4. Cetuximab Resistance Mechanisms and Potential for RAC1/RAC1B to Contribute to Cetuximab Resistance

### 4.1. EGFR Signaling

**Figure 3:**
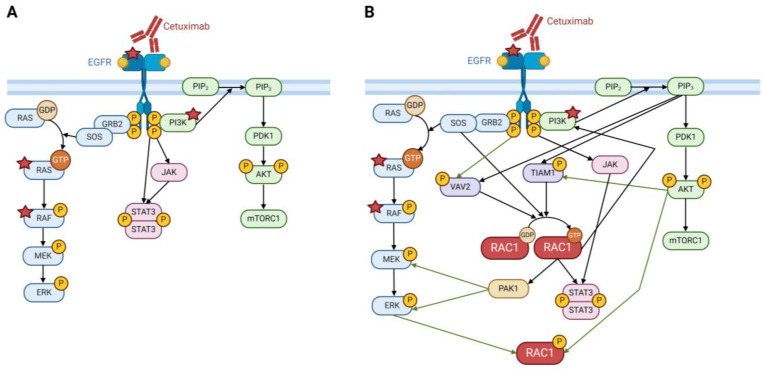
Constitutive pathway activation as a mechanism of cetuximab resistance and how RAC1 interacts with these pathways. (**A**) Schematic of canonical pathways activated by EGFR including RAS/RAF/MEK/ERK, PI3K/AKT, and JAK/STAT pathways. Red stars indicate activating mutations that have been found to confer resistance to cetuximab. (**B**) Schematic of RAC1 interactions with the MAPK, PI3K, and JAK/STAT signaling pathways activated by EGFR. Red stars indicate activating mutations that confer resistance to cetuximab and could also activate RAC1 to contribute to cetuximab resistance. Green arrows indicate activity through phosphorylation, and black arrows indicate activation through other mechanisms with respect to RAC1 activation. The EGFR effector SOS can act as a GEF for RAC1 and increase RAC1-GTP levels. Activated RAC1 activates its downstream effector PAK1, which can phosphorylate and activate both MEK and ERK. Activated ERK can phosphorylate RAC1, which inactivates the GTPase activity. Phosphorylated EGFR can directly phosphorylate and activate VAV2, which activates RAC1 [121,122]. The PI3K product PIP3 binds to VAV2 and TIAM1 to induce a conformational change that increases their affinity for RAC1. [123] Activation of AKT downstream of PI3K leads to TIAM1 phosphorylation and activation, and AKT can phosphorylate RAC1, which inactivates RAC1 [124]. Furthermore, RAC1 can activate mTORC1/mTORC2, and inhibition of mTORC1/mTORC2 decreases RAC1 activity, although the mechanism is unclear [125,126]. Figure created in Biorender.com.

EGFR activation occurs when ligand binding to EGFR induces receptor dimerization and subsequent autophosphorylation. Phosphorylation of the kinase domain activates downstream effector molecules either directly or through adaptor molecules. The pathways activated by EGFR include Ras/MAPK, phosphatidylinositol 3-kinase (PI3K)/AKT, and signal transducers and activators of transcription (STAT) pathways to increase cell proliferation, cell growth, and invasion and migration (Figure 3A) [43,116]. These pathways can become activated independent from EGFR signaling through mutations in downstream pathway components in cetuximab-resistant CRC, but it is possible that RAC1 overexpression could also contribute to pathway activation, as RAC1 interacts with many EGFR signaling effectors.

RAC1 has been shown to activate the Ras/MAPK, PI3K/AKT, and STAT signaling pathways, and these pathways in turn also activate RAC1 (Figure 3B) [102,103]. Figure 3B highlights the known interactions between EGFR signaling components and proteins involved in RAC1 signaling pathways. Briefly, the Ras GEF son of sevenless (SOS) also acts as a GEF for RAC1, and activated RAC1 can increase the phosphorylation of MEK and ERK through the effector PAK1 [117,118]. The activation of RAC1-ERK signaling increases CRC cell migration and invasion [119]. Activated ERK can in turn phosphorylate RAC1, resulting in increased nuclear RAC1 that can alter gene transcriptional programs to promote cell proliferation [54]. Furthermore, RAC1 can activate STAT3, which is a downstream effector of EGFR signaling, and STAT3 activation via RAC1 signaling increases cell migration [120].

In addition to MAPK pathway interactions, studies investigating RAC1 activation following EGF stimulation showed that PI3K activity can increase levels of RAC1-GTP in intestinal epithelial cells, and RAC1-GTP can in turn activate PI3K [73,102,127]. PI3K increases RAC1-GTP levels by activating RAC1 GEFs such as TIAM1 and VAV2 through the PI3K product phosphatidylinositol (3,4,5)-triphosphate (PIP_3_), which can increase the affinity of the GEFs for RAC1 [61,123,127,128]. GEFs can also be phosphorylated, which also increases the affinity for RAC1, and this can occur through AKT activity or after binding to phosphorylated EGFR [121,129,130]. In CRC cell lines, increased EGFR activation leads to increased activation and accumulation of TIAM1 [129]. TIAM1 is found to be overexpressed in CRC, which could be in response to increased EGFR activation, or alternatively, in response to WNT signaling, as TIAM1 is a WNT responsive gene [131,132]. The treatment of lung cancer cell lines with cetuximab resulted in decreased expression of TIAM1 and reduced RAC1 activation [129]. The re-expression of TIAM1 through WNT signaling or constitutive activation of AKT can contribute to RAC1 activation and cetuximab resistance. Therefore, the treatment of cetuximab-resistant CRC tumors with TIAM1-RAC1 interaction inhibitors could prove to be a viable combination therapy option to reduce CRC progression.

While RAC1 has many overlapping pathways with EGFR that could contribute to cetuximab resistance, there is less extensive evidence for RAC1B interactions with EGFR downstream effector proteins. The studies outlining RAC1 interactions with EGFR signaling described above need to be investigated with RAC1B to determine if similar pathways are involved in RAC1B effector functions. In support of the potential for RAC1B to contribute to cetuximab resistance, BRAF(V600E) mutations commonly seen in CRC resistance to cetuximab generally induce senescence in CRC, but if colonocytes express BRAF(V600E) and have RAC1B overexpression, senescence markers are downregulated and cells have a growth advantage [40,133]. Additionally, RAC1B can reduce apoptosis through the activation of AKT2, a downstream effector of EGFR [87]. Although the more recent studies suggest that RAC1B can interact with EGFR directly and RAC1B is needed for EGFR phosphorylation, follow up studies and verification in other models have not been completed [94]. This relationship between EGFR and RAC1B has been implicated in cetuximab resistance, where investigators treated cetuximab-resistant patient-derived organoids with cetuximab and an exon 3b targeting antisense oligonucleotide that prevents RAC1B expression, and they found that decreasing RAC1B expression restores cetuximab sensitivity, suggesting that RAC1B could play a role in cetuximab resistance [94]. However, the mechanism behind this observed response is unclear and has yet to be elucidated.

### 4.2. Activation by Other Receptor Tyrosine Kinases

Many other RTKs activate similar downstream signaling pathways to EGFR, including MAPK and PI3K/AKT pathways. RTKs that have been found to activate these pathways independent from EGFR activation in CRC that can confer resistance to cetuximab include ERBB2/HER2, mesenchymal–epithelial transition factor (MET) receptor, and insulin-like growth factor receptor (IGF-1R) [134,135,136,137,138,139,140]. The activation of other RTKs in response to cetuximab treatment can be due to increased receptor expression, increased ligand expression, or, more recently described, altered ligand processing [141,142]. For example, overexpression of the MET ligand hepatocyte growth factor (HGF) in CRC three-dimensional (3D) collagen cell culture colonies leads to cetuximab resistance [142]. RAC1 GEFs can be activated by RTKs including MET and ERBB2 [61]. The activation of MET through its ligand HGF activates RAC1 to promote the migration and invasion of cells [143,144]. This suggests that MET activation through either receptor or HGF overexpression in cetuximab-resistant CRC could activate RAC1, perpetuating CRC progression and cetuximab resistance. ERBB2 targeted therapies are widely used to treat breast cancer and are being investigated for use in CRC, and there are multiple trials investigating the efficacy of ERBB2-targeted therapies in metastatic CRC [135,145,146,147]. Interestingly, increased RAC1 activity in breast cancer confers resistance to the HER2-targeting antibody trastuzumab by altering cell morphology and misregulating the levels of HER2 receptor on the cell surface [148].

### 4.3. EGFR Expression and Activation

Another mechanism of resistance against cetuximab is altered levels of EGFR in the cells following cetuximab treatment. Internalized EGFR can either be recycled and returned to the membrane or be degraded in the lysosome. Cetuximab treatment leads to receptor internalization and subsequent degradation, and while the exact levels of EGFR on the cell surface do not predict cetuximab response, more recent studies suggest that the destination of the receptor following internalization better predicts the response [149,150]. One study found that in response to cetuximab treatment, CRC cell lines can become resistant by decreasing the cell surface expression of EGFR by increasing receptor ubiquitination and degradation [151]. In line with increased EGFR degradation, in vivo models showed that the loss of RAC1B expression leads to increased trafficking of EGFR to the lysosome, which leads to subsequent degradation, thus decreasing EGFR levels on the plasma membrane and signal transduction [94]. Alteration of the amount of receptor on the membrane changes the sensitivity of cells to receptor-targeted therapy. By modulating receptor levels on the cell surface, RAC1B could influence cetuximab sensitivity.

The involvement of RAC1 and RAC1B in EGFR signaling and downstream component activation suggests that RAC1 and RAC1B overexpression could be a form of resistance to cetuximab through constitutive signal activation. This has not yet been directly shown, but the evidence described above provides a basis to predict that RAC1 and RAC1B could contribute to cetuximab resistance through this common resistance mechanism. Targeting RAC1 and RAC1B either directly or through GEF regulation could reduce signal activation independent from EGFR and help restore sensitivity to cetuximab if these proteins are overexpressed.

### 4.4. Epithelial-to-Mesenchymal Transition

The epithelial-to-mesenchymal transition (EMT) is a process that enables the invasion and metastasis of cancer cells, a well-known hallmark of cancer and driver of cancer-related deaths [3,152]. EMT in CRC can be initiated by increased WNT signaling, which increases expression of the transcription factor Snail, which then activates EMT-promoting genes [153]. Additionally, high EGFR signaling can promote EMT [154].

EMT in solid tumors is associated with resistance to both chemotherapy and targeted therapy. Although it has been difficult to directly describe the mechanisms and the role of EMT in CRC resistance to cetuximab, there is some evidence of a relationship between these two phenotypes. Studies have shown that there is a correlation between the epithelial marker E-cadherin and sensitivity to EGFR inhibition, where cells expressing E-cadherin are more sensitive to EGFR inhibition; on the other hand, CRC cell lines expressing mesenchymal markers such as Zeb1, vimentin, and Snail are insensitive to EGFR inhibitors [155]. Moreover, in head and neck cancer patients, there is increased expression of EMT markers such as Twist, Zeb1, and Lef1 in post-cetuximab treatment tumor biopsies, suggesting EMT is likely a form of acquired resistance [156]. EMT has also been shown to be a form of acquired resistance to EGFR small molecule inhibitors in lung cancer [157].

During EMT, aspects of cell morphology such as cell shape and distribution of actin in the cell change, and cell morphology has been shown to be a predictor of therapy response and resistance. For example, CRC cell lines grown in 3D collagen cultures can show two different phenotypes, one with organized cysts of polarized cells (cystic colonies) and one with solid masses of disorganized cells with protrusions (spiky colonies) [158]. The cystic colonies maintain apico-basolateral polarity, while the spiky colonies lack the tight junctions and adherens junctions associated with epithelial phenotypes [158,159]. The overexpression of HGF that results in cetuximab resistance, as mentioned above, can also induce a switch from cystic to spiky colonies that is associated with EMT [142]. Spiky colonies also have an enriched gene signature associated with EMT. Additionally, the disorganized mesenchymal colonies are more resistant to cetuximab compared to the cystic colonies [159]. This suggests a relationship between tumor cell morphology, EMT, and cetuximab resistance. These changes in morphology associated with EMT and drug resistance could be associated with RAC1.

EMT requires cytoskeletal reorganization that directs changes in cell shape and motility, which can be regulated by RAC1 through PAK1, which subsequently activates LIM kinase 1 (LIMK1) and decreases cofilin activity to stimulate actin polymerization [45]. One group found that the increase in migration and invasive capabilities of CRC cells was due to regulation of the RAC1-PAK1-LIMK1-cofilin pathway regulating actin polymerization [160,161]. The deletion of RAC1 in CRC LoVo cells has negative impacts on the migration and invasion capacity of these cells [162]. Given that EMT and migration lead to metastases, RAC1 overexpression is found in metastatic CRC tissues and can promote CRC cell migration and invasion [79]. The overexpression of PAK1 can also contribute to cancer progression and metastasis [163]. RAC1 expression has been shown to be higher in liver metastases compared to primary CRC tumor cells, and inhibiting RAC1 decreases liver metastasis [164]. Furthermore, hypoxia, a known inducer of EMT, can increase EGFR signaling that facilitates cell migration and decreased adhesion through RAC1 activity [165]. Hypoxia is also associated with RHO GTPase overexpression, and RAC1 stabilizes factors that regulate hypoxia, such as hypoxia-inducible factor 1α, thus promoting hypoxia and facilitating EMT [166]. Tissues from patients with high stage disease, increased spread to lymph nodes, and metastases also had RAC1 overexpression that promoted EMT in colorectal cancer through the activation of STAT3 [120]. While RAC1 has not been shown to be involved in EMT in response to cetuximab treatment and colorectal cancer, studies in breast cancer have shown that RAC1 contributes to resistance to the HER2 inhibitor trastuzumab, and this resistance is associated with alterations in cell morphology that impair trastuzumab-mediated endocytic downregulation of HER2 driven by RAC1 [148]. Interestingly, in breast cancer, the ratio of RAC1 and RAC1B can be used to determine if the tumor cells have a more epithelial or mesenchymal phenotype, where cells expressing more RAC1B have more epithelial properties and RAC1 expression is associated with a mesenchymal phenotype [167].

RAC1B facilitates EMT progression following matrix metalloproteinase 3 (MMP-3) expression in breast tumors by increasing ROS that induce the expression of EMT transcription factor Snail [99]. Given that CRC EMT is driven by Snail expression, the induction of RAC1B expression could facilitate this pathway as well [153]. In breast cancer, RAC1B was found to primarily localize to the membrane and stimulate production of ROS when cells are in a stiff microenvironment that also stimulates EMT [100]. Additionally, RAC1B expression negatively regulates the expression of E-cadherin in CRC cell lines, so RAC1B further facilitates EMT by actively decreasing cell adhesion [56].

Overall, EMT is a loosely defined mechanism of resistance to cetuximab in CRC, but inferences can be made to relate the functions of RAC1, RAC1B, and their contributions to EMT that could contribute to therapy resistance based on evidence from other cancers and CRC. RAC1 plays more of a driving role in EMT by the regulation of cell signaling, cell morphology, and ultimately, movement. On the other hand, RAC1B is more of a byproduct of EMT initiation that helps further progress EMT and facilitates cell survival during the process. Currently, EMT is not directly targeted by specific treatments in CRC, but given the roles of RAC1 and RAC1B in EMT described above, RAC1 and RAC1B could provide an option for combination therapies since EMT is associated with treatment resistance.

### 4.5. CRC Clonal Molecular Subtype and CMS Switching

CRC tumors with similar characteristics can be grouped into various subtypes known as consensus molecular subtypes (CMS), with CMS4 representing an EMT phenotype and transcriptional signature [168]. CMS1 tumors are hypermutated and microsatellite instable, CMS2 tumors are “canonical” tumors with epithelial characteristics and WNT signaling, and CMS3 tumors have altered metabolic profiles. Recent studies have found that CMS2 tumors respond best to cetuximab either alone or in combination with chemotherapy [169,170]. Tumors with CMS4 characteristics have been associated with an EMT transcriptional signature, as well as chemotherapy resistance [171]. One group suggests that CMS-switching from CMS2 to CMS4 can be a resistance mechanism to cetuximab, providing further evidence that EMT could be an acquired cetuximab resistance mechanism [172].

This switch from cetuximab-sensitive CMS2 tumors to cetuximab-resistant CMS4 tumors could be driven by RAC1B. One group found that high RAC1B expression is significantly associated with CMS2 tumors and can be found in CMS4 tumors, and RAC1B is associated with worse patient outcomes according to TCGA data [94]. The authors also discuss an interesting conflict as CMS2 tumors typically respond better to treatment, but CMS2 tumors could be divided into RAC1B-low and RAC1B-high tumors, and RAC1B high CMS2 tumors have similarly low patient outcomes as CMS4 tumors [94,168]. Thus, inhibiting RAC1B in CMS2 tumors with high RAC1B expression in combination with cetuximab could be a treatment option that prevents CMS switching to cetuximab-resistant CMS4 subtypes. Investigating this phenomenon further could help provide greater insight into the drivers of cetuximab resistance mechanisms. CMS switching is a unique feature of CRC and warrants further study, especially with respect to treatment resistance, RAC1, and RAC1B.

## 5. Conclusions and Future Directions

RAC1 and RAC1B have been proposed as potential targets for cancer therapies since both can play a role in many different aspects of tumor biology and therapy resistance [45]. However, the evidence for RAC1 and RAC1B in CRC therapy resistance is weak with only a few publications showing limited evidence for RAC1 and RAC1B in CRC chemotherapy resistance and one experiment relating RAC1B to cetuximab resistance. Despite this, we propose that RAC1 and RAC1B could contribute to cetuximab resistance in CRC based on the known pathways that RAC1 and RAC1B activate that are related to known cetuximab resistance mechanisms.

Given the important roles of RAC1 and RAC1B in CRC tumor biology and progression described in this review, inhibiting RAC1 and RAC1B is a potential therapeutic option. There are multiple avenues for RAC1/RAC1B inhibition, including GTPase-GEF interaction inhibition, GTP-binding inhibition, and preventing binding to downstream effector molecules, and small molecule inhibitors have been shown to be effective at inhibiting RAC1 activity in vitro [51,173]. For example, the first RAC1-specific inhibitor, called NSC23766, works by inhibiting RAC1 interactions with the GEFs TIAM1 and TRIO [174]. Unfortunately, many of the currently available RAC1 inhibitors including NSC23766 are not clinically viable as the effective doses are high, where NSC23766 has an IC_50_ value between 50 µM and 75 µM [174]. Additionally, RAC1 is ubiquitously expressed, so there is an increased chance of off-target effects and unwanted side effects. Recently, work is being carried out to generate more effective derivatives of inhibitors like NSC23766, such as EHop-016 and MBQ-167 with lower IC_50_ values (around 100 and 78 nM, respectively) and more targeted inhibition [175,176,177]. Despite RAC1B being more active and seemingly more relevant for CRC pathology, there are a limited number of inhibitors that have been found to be effective against RAC1B in addition to RAC1. One small molecule called EHT 1864 has been shown to inhibit both RAC1 and RAC1B with increased potency (IC50 is around 5 µM), but there is limited evidence for use of EHT 1864 in CRC [178]. However, in mouse models, both NSC23766 and EHT 1864 were shown to have negative effects on platelets at their effective concentrations, highlighting the off-target and negative side effects of these drugs and their limitations for use in humans and clinical trials [179].

**Figure 4:**
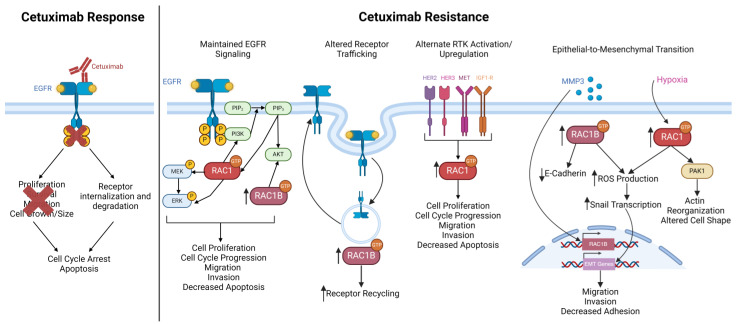
Cetuximab mechanism of action and summary of how RAC1 and RAC1B could contribute to the known mechanisms of cetuximab resistance in CRC. Treatment with cetuximab prevents the activation of EGFR kinase domains, preventing receptor phosphorylation and activation of downstream signaling cascades. Cetuximab also promotes the internalization and degradation of the receptor, and together, these mechanisms lead to cell-cycle arrest and apoptosis. Alternatively, RAC1 and RAC1B can contribute to known cetuximab resistance mechanisms through activation by other RTKs, and by perpetuating downstream EGFR signaling pathways, promoting receptor recycling to the membrane following internalization and promoting the epithelial-to-mesenchymal transition. Figure created with Biorender.com.

Furthermore, inhibiting RAC1 can restore sensitivity to targeted therapy trastuzumab in breast cancer, suggesting that RAC1 inhibition could be an additional target for therapy in CRC [148]. This effect further strengthens the relationship between RAC1 and EGFR signaling pathways and provides a potential alternative therapy for patients with resistant tumors. Future directions for this field include developing more potent RAC1 and RAC1B inhibitors, leading to future clinical trials that may benefit patients with RAC1 and RAC1B overexpressing tumors. Given that the recent literature investigating RAC1 and RAC1B in CRC suggests a strong interaction with the immune microenvironment, more research into combination therapies with immunotherapy and cetuximab is needed. This research would also provide a basis for inhibiting RAC1 and RAC1B in combination with cetuximab to prevent and manage cetuximab resistance. Cetuximab resistance remains a significant obstacle in treating CRC, and it is necessary to investigate all possible mechanisms of resistance to develop combination therapies that can prevent or be used in response to resistance. The activation of RAC1 and RAC1B represents one of many pathways that are activated in CRC, and there are many parallel pathways that could contribute to the overall incidence of CRC cetuximab resistance. While future studies directly testing the relationship between RAC1 and RAC1B and cetuximab resistance in CRC are needed, the evidence summarized in this review provides strong support for a role for RAC1 and RAC1B in intrinsic and acquired resistance to cetuximab (Figure 4).

## Figures and Tables

**Table 1:** Comparison of RAC1 and RAC1B protein features and biochemical characteristics.

Feature	RAC1	RAC1B	Refs.
Protein Length (amino acids)	192	211	[52,57,58]
Post-translational modifications	Ubiquitination: K147 and K166 Phosphorylation: S71, T108, and Y64 Geranylgeranylation: CAAX motif (AA 189–192)	Minimal ubiquitination Phosphorylation: unknown Geranylgeranylation: CAAX motif (AA 208–211)	[54,55,59,60]
GTPase Dynamics	GEF-dependent activation Regulated by RHO-GDIs Regulated by GAPs	GEF-independent activation Not regulated by RHO-GDIs Regulated by GAPs Impaired GTP hydrolysis Decreased GDP affinity Increased flexibility between switch I and switch II domains	[53,61,62,63,64]
Predominant State at Baseline	GDP-bound	GTP-bound	[62]
Protein Expression	Ubiquitous	Almost exclusively expressed in tumor tissue (breast, colon, lung)	[57,58]
Cellular Localization	Plasma Membrane Cytoplasm Nucleus	Plasma Membrane (predominantly) Nucleus	[53,56]
Splicing Factors that Favor Isoform Expression	SRSF3 hnRNP A1	SRSF1 ESRP1	[65,66,67,68,69,70]
Signaling Pathways Regulating Splicing Factor Expression and Activity	WNT/β-catenin/TCF4	EGFR/PI3K/AKT MMP3 IL-6/STAT3	[65,67,68,69]
Downstream Effector Proteins and Pathways	PAK1, WAVE, JNK, IQGAP1, RelB-NF-κB2/p100, β-catenin/TCF4, NOX1, EGFR and EGFR downstream signaling proteins	NOX1, SmgGDS, RACK1, p120(ctn), disheveled/ β-catenin, NF-κB, EGFR and EGFR downstream signaling proteins (MEK, ERK)	[45,46,47,50]

## Abbreviations

CRC	Colorectal cancer
GTPase	guanosine triphosphatase
5-FU	5-fluorouracil
EGFR	epidermal growth factor receptor
VEGF	vascular endothelial growth factor
RTK	receptor tyrosine kinase
FDA	Food and Drug Administration
KRAS	Kirsten rat sarcoma viral oncogene homolog
BRAF	v-raf murine sarcoma viral oncogene homolog B1
PI3K	phosphatidylinositol-4,5-bisphosphate 3-kinase
PIK3CA	phosphatidylinositol-4,5-bisphosphate 3-kinase catalytic subunit alpha
EMT	epithelial-to-mesenchymal transition
RAC1	Rac family small GTPase 1, Ras-related C3 botulinum toxin substrate 1
GDP	guanosine diphosphate
GTP	guanosine triphosphate
GEF	guanine nucleotide exchange factor
GAP	GTPase activating proteins
GDI	guanine nucleotide-dissociation inhibitors
SRSF1	serine/arginine rich splicing factor 1, SF/AF2
SRp20	serine/arginine rich protein 20, SRSF3
EGF	epidermal growth factor
hnRNP A1	heterogeneous nuclear ribonucleoprotein A1
ESRP1	epithelial splicing regulatory protein 1
LGR5	leucine-rich repeat-containing G protein-coupled receptor 5
APC	adenomatous polyposis coli
ROS	reactive oxygen species
SOX9	SRY-box transcription factor 9
IQGAP1	IQ motif-containing GTPase-activating protein 1
NOX1	NADPH oxidase 1
FOLFOX	leucovorin calcium (folinic acid), fluorouracil, and oxaliplatin
XELOX	capecitabine and oxaliplatin
TCGA	The Cancer Genome Atlas
PAK1	p21 activated kinase
RACK1	receptor for activated kinase 1
STAT	signal transducers and activators of transcription
SOS	son of sevenless
PIP3	phosphatidylinositol (3,4,5)-triphosphate
MET	Mesenchymal–epithelial transition factor receptor
IGF-1R	insulin-like growth factor receptor
HGF	hepatocyte growth factor
3D	three dimensional
LIMK	LIM kinase 1
MMP-3	matrix metalloproteinase 3
CMS	consensus molecular subtypes
